# Iliopsoas hydatid cyst mimicking an ovarian tumor: a diagnostic pitfall in an endemic setting—a case report

**DOI:** 10.1186/s12879-026-13638-3

**Published:** 2026-05-22

**Authors:** Melkamu Siferih, Tesfaye Negasa, Abebaw Welelaw

**Affiliations:** 1 Department of Obstetrics and Gynecology, School of Medicine, Debre Markos University, Debre Markos, Ethiopia; 2 Department of General Surgery, Medawolabu General Hospital, Dodola, Oromia Region Ethiopia; 3 Department of General Surgery, School of Medicine, Debre Markos University, Debre Markos, Ethiopia

**Keywords:** Iliopsoas hydatid cyst, Ovarian tumor mimic, Diagnostic pitfall, Endemic setting, Case report, Ethiopia

## Abstract

**Background:**

Cystic echinococcosis is endemic in Ethiopia. Although the liver and lungs are most commonly affected, primary involvement of the iliopsoas muscle is rare and may closely mimic gynecologic pathologies, leading to diagnostic delay or mismanagement.

**Case presentation:**

We report the case of an 18-year-old Ethiopian woman who presented with a two-year history of progressively worsening lower abdominal pain and a palpable right lower abdominal mass. Initial ultrasonography suggested a right adnexal cystic lesion. Subsequent computed tomography demonstrated a well-defined cystic mass adjacent to the right iliopsoas muscle. Surgical exploration through a right lower abdominal oblique (Modified Rutherford-Morrison) incision revealed a **10 × 8** cm hydatid cyst arising from the iliopsoas muscle; the dimensions were confirmed intraoperatively. The cyst contained a dominant daughter cyst and multiple smaller daughter cysts. Histopathological examination confirmed cystic echinococcosis. The patient underwent complete pericystectomy followed by albendazole therapy, with uneventful postoperative recovery.

**Conclusion:**

This case highlights the diagnostic challenge posed by hydatid disease in unusual anatomical locations, where it can closely mimic gynecologic or neoplastic pathologies. In endemic settings, timely diagnosis requires heightened clinical suspicion and appropriate imaging, while optimal outcomes depend on coordinated multidisciplinary management integrating definitive surgery and antiparasitic therapy.

## Introduction

Cystic echinococcosis (CE), caused by *Echinococcus granulosus*, remains a major zoonotic disease and public health concern in endemic regions, including Ethiopia, where close human-livestock interaction facilitates ongoing transmission [1–3]. The liver and lungs are the most commonly affected organs; however, hydatid cysts may occasionally occur in atypical locations such as muscle, bone, and retroperitoneal structures, often leading to diagnostic difficulty and delayed management [4–6]. Primary involvement of the iliopsoas muscle is rare, accounting for fewer than 3% of reported cases of CE, musculoskeletal involvement representing an uncommon manifestation of the disease in general [7, 8]. When present, such cysts may closely mimic neoplastic, infectious, or gynecologic conditions—including ovarian tumors or adnexal masses—thereby posing a significant diagnostic challenge, particularly in young women [9–11]. This difficulty is further compounded in resource-limited settings, where access to advanced imaging modalities and serologic confirmation may be restricted [12–14].

Although CE is endemic in Ethiopia due to widespread pastoral and livestock practices, musculoskeletal and retroperitoneal involvement—including iliopsoas localization—remains exceedingly rare, with only isolated case reports described in endemic regions [15, 16]. Consequently, atypical presentations may be overlooked, increasing the risk of delayed diagnosis, inappropriate management, and serious complications such as cyst rupture, secondary dissemination, or anaphylactic reactions [17–19].

Here, we report a rare case of a primary iliopsoas hydatid cyst in an Ethiopian woman that radiologically mimicked an ovarian tumor. This case underscores the importance of maintaining a broad differential diagnosis in endemic settings and highlights the role of multidisciplinary collaboration in achieving accurate diagnosis and optimal management.

## Case presentation

An 18-year-old Ethiopian woman (para 1) presented in June 2024 with a two-year history of progressively worsening right lower abdominal pain and swelling. The pain was described as dull, intermittent, non-radiating, and gradually increasing in intensity; however, a formal numerical pain scoring system was not applied. She denied weight loss and urinary symptoms such as dysuria, frequency, urgency, or hematuria. Current body weight was measured at admission, with no evidence of clinically significant weight loss, although previous baseline weight records were not available for comparison. Her menstrual cycles were regular, with no abnormal vaginal bleeding or gynecologic symptoms.

She had no significant past medical or surgical history, including tuberculosis, malignancy, pelvic inflammatory disease, or prior abdominal surgery. Her obstetric history included one uncomplicated term vaginal delivery. There was no family history of malignancy. She reported close contact with domestic animals, including dogs and sheep, and residence in a rural livestock-farming area.

On physical examination, she was well-appearing, alert, and in no acute distress. Her vital signs were stable, with a blood pressure of 110/70 mmHg, pulse rate of 82 bpm, respiratory rate of 18 breaths per minute, temperature of 36.7 °C, and oxygen saturation of 98% on room air. Abdominal examination revealed a firm, globular, non-mobile, non-tender mass in the right lower abdomen, measuring approximately **10 × 8** cm. There was no rebound tenderness, guarding, or rigidity, and the liver and spleen were not enlarged. There was also no shifting dullness or fluid thrill, suggesting the absence of ascites. On auscultation, bowel sounds were normal, and a gynecologic examination showed normal external genitalia with no lesions or abnormal discharge. Upon bimanual examination, it was difficult to separate the mass from the ovary or the uterus. The cervix was firm, non-tender, with no cervical motion tenderness. No adnexal or cervical motion tenderness was elicited.

The patient had no clinical features suggestive of hepatic or pulmonary involvement, including the absence of right upper quadrant pain, jaundice, cough, chest pain, dyspnea, or hemoptysis. Abdominopelvic ultrasonography did not reveal any cystic lesions in the liver or other intra-abdominal organs. Furthermore, computed tomography showed no hepatic involvement, and the visualized lung bases were unremarkable. Dedicated chest imaging was not performed due to the absence of respiratory symptoms and resource considerations.

Given the patient’s age, the right lower abdominal location of the mass, and the inability to clearly delineate the mass from the ovary or uterus on bimanual examination, the initial clinical impression was a right adnexal mass.

Laboratory tests, including complete blood count (CBC), liver function tests, renal function tests, and random blood sugar (RBS), were all within normal limits, and no eosinophilia was noted. Abdominopelvic ultrasonography revealed a well-defined, anechoic, double-walled cystic mass (**9 × 8** cm) with posterior acoustic enhancement, located in the right adnexal region.

**Fig. 1:**
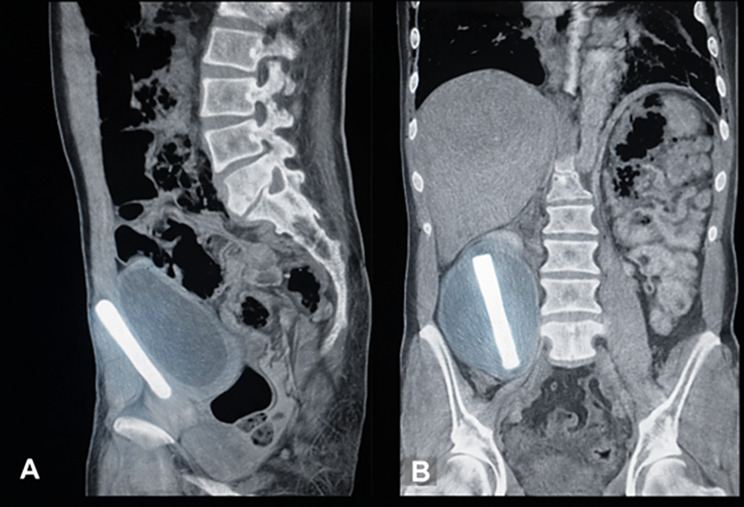
A non-contrast computed tomography image demonstrating a hydatid cyst located in the right iliopsoas region. (**A**) Sagittal reconstruction showing the cyst in the right iliac fossa extending with in the right iliac fossa. (**B**) Coronal reconstruction illustrating the relationship of the cyst to the psoas muscle, iliac bone, and adjacent pelvic structures

A contrast-free abdominopelvic CT scan confirmed a well-defined cystic mass (**5.9 × 8.2 × 9.1**cm) with near-water attenuation and a thin, perceptible wall, with no septations, solid components, or calcifications. The mass was situated lateral to the right psoas muscle, pushing it medially, and was inferior to the cecum. It was partially attached to the iliac bone without bony erosion, and clear fat planes separated it from the external iliac vessels (Fig. 1).

**Fig. 2:**
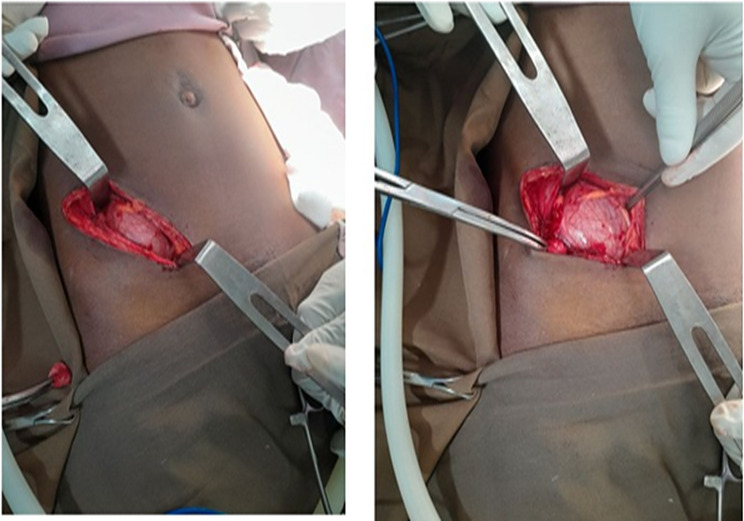
Intraoperative photographs of an iliopsoas muscle hydatid cyst immediately prior to enucleation via a modified Rutherford-Morrison incision

**Fig. 3:**
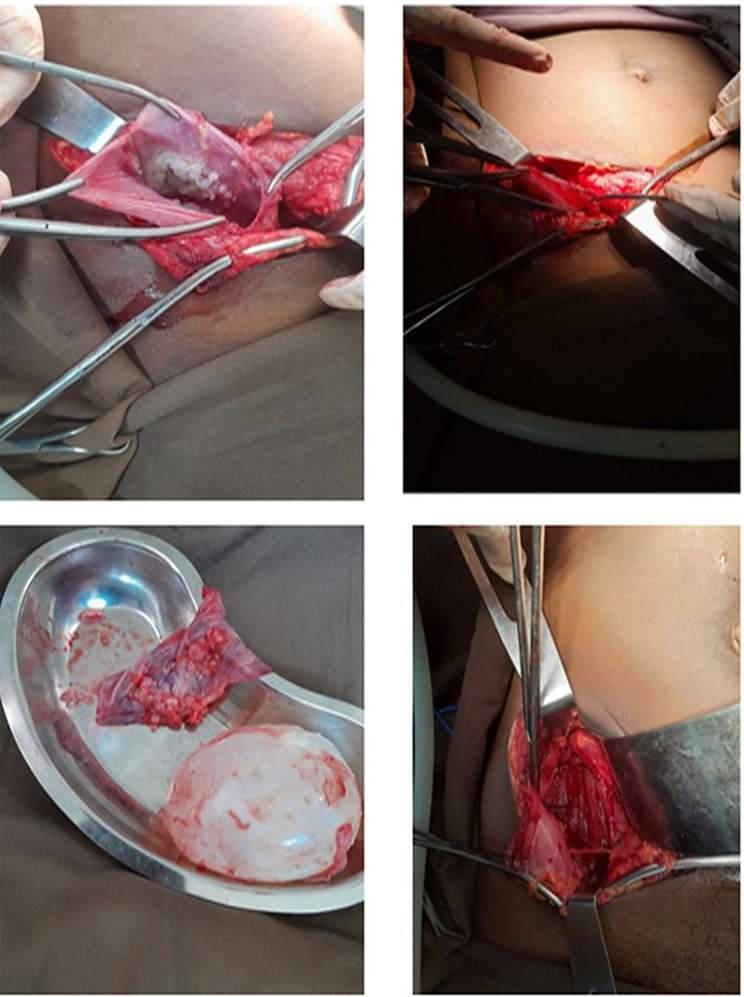
Detailed intraoperative view of the hydatid cyst and its pericystic planes during pericystectomy

Given the imaging finding, the differential diagnoses included a right ovarian simple cyst, mesenteric cyst, and hydatid cyst. Surgical exploration was performed via a right lower abdominal oblique (Modified Rutherford-Morrison/iliac fossa) incision, revealing a **10 × 8** cm cystic mass in the right iliac fossa, located between the psoas and iliacus muscles. The cyst had a well-formed capsule and contained a single large daughter cyst (6 × 8 cm) along with multiple smaller daughter cysts within the ectocyst. Intraoperative precautions were taken to prevent intraoperative spillage, including careful isolation of the operative field with saline-soaked gauze pads, and meticulous handling of the cyst to avoid rupture. The operative field was irrigated with normal saline. A complete pericystectomy was successfully performed. The presence of multiple daughter cysts is consistent with an active stage of cystic echinococcosis stage CE2 according to the WHO-IWGE classification. Although primarily developed for hepatic disease, this framework can be applied to extrahepatic sites to support staging and management. The variation in cyst dimensions across modalities reflects differences in imaging planes and the definitive intraoperative measurements (Figs. 2 and 3).

Histopathological examination confirmed hydatid disease, showing daughter hydatid cysts with a laminated membrane. The patient had an uneventful postoperative course with no intraoperative or early postoperative complications such as cyst spillage, wound infection, bleeding, or anaphylactic reaction. The patient was discharged in stable condition on albendazole 400 mg PO BID for one month (Body weight at admission: 55 kg; 15 mg/kg/day in two divided doses), and scheduled follow-up surveillance to monitor for recurrence or later complications. Medium- and long-term follow-up data are not yet available.

## Discussion

Hydatid disease remains a significant diagnostic and therapeutic challenge, particularly when it occurs in unusual anatomical locations such as the iliopsoas compartment. While Echinococcus granulosus predominantly affects the liver and lungs, musculoskeletal involvement is uncommon, accounting for a smaller proportion of cases due to the relative unfavorable environment of skeletal muscle for larval growth, which is attributed to factors such as constant contractility, high lactic acid concentration, and limited local blood flow. When it does occur, it often arises in deep-seated locations such as the iliopsoas muscle, where it may remain clinically silent for prolonged periods [5, 20, 21].

In our case, the deep retroperitoneal location led to significant diagnostic difficulty, as the lesion closely mimicked intra-abdominal or gynecologic pathologies, particularly an ovarian mass on initial evaluation. This case highlights that accurate assessment of lesion location is more critical than relying solely on organ-based interpretation, particularly in deep pelvic and retroperitoneal masses, where anatomical distortion may obscure the true origin of the lesion. The close anatomical relationship between the iliopsoas compartment, adnexal structures, and pelvic sidewalls may further obscure the true site of origin of deep retroperitoneal cystic lesions, particularly on ultrasonography where tissue planes are less clearly delineated. This overlap represents a major diagnostic pitfall in musculoskeletal hydatid disease, where atypical anatomical positioning and non-specific imaging features may delay correct diagnosis. Our case, therefore, stresses the importance of maintaining a high index of suspicion for hydatid disease in endemic regions, even in uncommon musculoskeletal sites, and integrating cross-sectional imaging with clinical context to avoid misdiagnosis.

The diagnosis in our case followed a multidisciplinary pathway involving gynecology, radiology, and general surgery, with initial misinterpretation as a complex ovarian cyst on ultrasound and subsequent CT suggesting a retroperitoneal/iliopsoas origin. Definitive diagnosis was delayed by the absence of pathognomonic imaging features and lack of serologic confirmation, and was ultimately established intraoperatively during complete pericystectomy.

Ultrasonography is a valuable first-line tool for hydatid disease, but it has limitations in deep and atypical locations. Imaging with CT or MRI enhances diagnostic accuracy by delineating cyst morphology, location, and relation to adjacent structures [22, 23, 24]. In this case, non-contrast CT revealed a thin-walled, near-water density cyst adjacent to the psoas muscle, which, although characteristic of hydatid disease, was initially misinterpreted due to its uncommon location. Definitive diagnosis was confirmed via surgical exploration and histopathology, demonstrating daughter cysts and laminated ectocyst—hallmarks of hydatid disease [25–27]. CE is classified by the WHO-IWGE into active, transitional, and inactive stages based on cyst morphology [28, 29]. In our case, the multivesicular nature of the cyst corresponds to an active CE2 stage. Although this classification is primarily designed for hepatic lesions, it remains conceptually applicable to atypical sites such as the iliopsoas muscle, where diagnosis and staging are often more challenging [30, 31]. When compared with previously reported cases of iliopsoas or deep musculoskeletal hydatid cysts, the present case shares several features while also demonstrating important distinctions. Most reported cases involve adults in the third to fifth decades of life, with no consistent sex predominance reported, although some series show a slight male predominance [32–34]. Our patient falls within the typical age range but differs in sex, emphasizing that iliopsoas hydatid disease should be considered regardless of gender. Imaging findings in prior cases commonly describe well-defined cystic lesions adjacent to or involving the psoas muscle, occasionally with daughter cysts or calcifications on CT or MRI [8, 35]. In contrast, the lesion in our patient lacked overt pathognomonic features and was initially misinterpreted as a gynecologic pathology, reflecting a diagnostic challenge also described in cases near the adnexa or pelvic cavity [25–27, 36].

Surgical excision remains the mainstay of treatment for hydatid cysts in functionally critical or anatomically complex regions. Complete pericystectomy is preferred to minimize recurrence and prevent complications such as rupture or dissemination [37–39]. In this case, complete pericystectomy was achieved successfully. The surgical strategy employed in the present case aligns with current best practices and international recommendations [37, 40, 41]. Perioperative albendazole therapy further reduces relapse risk, with evidence supporting its use to improve cure rates and long-term outcomes. Albendazole is recommended to reduce the viability of protoscolices, limit secondary dissemination, and decrease recurrence risk, particularly in multivesicular cysts with daughter cysts. Although international guidelines recommend longer or cyclic therapy—typically 1–3 months preoperatively and 1–3 months postoperatively, particularly for deep soft tissue or complex cysts [42, 43].

In our case, the shorter regimen was an adaptation due to resource limitations and patient tolerance considerations, following complete surgical excision without intraoperative spillage. Preoperative albendazole was not administered because surgery was urgent and technically feasible. This approach aligns with current evidence indicating that perioperative benzimidazole therapy, even when abbreviated, can improve outcomes when combined with complete pericystectomy [44, 45]. Hydatid cysts evade host immunity through multiple mechanisms, including sequestration of complement regulatory proteins and modulation of cytokine signaling, which allow long-term persistence [46–49]. While these processes are not directly observable in routine clinical care, they may partly explain the often insidious and asymptomatic progression of cysts in atypical locations [50–52].

Advances in molecular diagnostics, including circulating parasite-derived microRNAs and novel biosensing technologies, hold potential for early detection, but their direct relevance to clinical management remains investigational [53–56].

This report is limited by its single-case design, lack of advanced serologic testing, absence of preoperative ultrasound documentation, and limited follow-up regarding recurrence and functional outcomes. In addition, standardized clinical monitoring parameters such as pain scale assessment and serial weight records were not systematically documented, which could have strengthened postoperative evaluation. Furthermore, formal patient-reported experience questionnaire was not performed.

Despite these limitations, the case highlights the diagnostic challenges of atypical hydatid cysts and emphasizes the importance of integrating clinical suspicion, imaging, surgical expertise, and histopathology in endemic settings. Optimal evaluation and management of such complex cases require a multidisciplinary approach involving radiology, surgery, pathology, and infectious disease specialists. The incorporation of echinococcosis-specific serologic testing, such as ELISA or indirect hemagglutination assay (IHA), where available, may further enhance diagnostic accuracy and reduce diagnostic delay, particularly in cases with inconclusive clinical and imaging findings. Future studies should adopt prospective designs with standardized assessments and longitudinal follow-up.

## Conclusion

Hydatid disease at atypical sites such as the iliopsoas muscle poses a significant diagnostic challenge due to overlap with more common abdominal, pelvic, and neoplastic conditions. This case reinforces the need for heightened clinical suspicion in endemic regions and the complementary role of CT and MRI in evaluating deep-seated lesions, while confirming that definitive diagnosis relies on histopathology. Complete surgical excision remains the cornerstone of management, with adjunctive albendazole therapy tailored to disease extent and completeness of resection. Importantly, optimal diagnosis and management require a multidisciplinary approach to reduce diagnostic delay and improve therapeutic accuracy. Greater awareness of such uncommon presentations is essential to improve outcomes in endemic settings.

## Data Availability

All data generated or analyzed during this study are included in this article.

## Abbreviations

CBC: Complete blood count
CE: Cystic echinococcosis
CE2: Cystic echinococcosis stage 2
CT: Computed tomography
ELISA: Enzyme-linked immunosorbent assay
IHA: Indirect hemagglutination assay
microRNAs: Micro ribonucleic acids
MRI: Magnetic resonance imaging
PO: Per OS, by mouth
RBS: Random blood sugar
WHO-IWGE: World Health Organization’s Informal Working Group on Echinococcosis
